# Different Respiratory Samples for COVID-19 Detection by Standard and Direct Quantitative RT-PCR: A Literature Review

**DOI:** 10.22037/ijpr.2021.115458.15383

**Published:** 2021

**Authors:** Maryam Ahmadzadeh, Hossein Vahidi, Arash Mahboubi, Fariba Hajifathaliha, Leila Nematollahi, Elham Mohit

**Affiliations:** a *Department of Pharmaceutical Biotechnology, School of Pharmacy, Shahid Beheshti University of Medical Sciences, Tehran, Iran. *; b *Department of Pharmaceutics, School of Pharmacy, Shahid Beheshti University of Medical Sciences, Tehran, Iran. *; c *Biotechnology Research Center, Pasteur Institute of Iran, Tehran, Iran.*

**Keywords:** COVID-19, Nasopharyngeal swab, Oropharyngeal swab, RT-qPCR, saliva, SARS-CoV-2, Sputum

## Abstract

The most common diagnostic method for detecting severe acute respiratory syndrome coronavirus 2 (SARS-CoV-2) infection is real-time quantitative reverse transcriptase-polymerase chain reaction (RT-qPCR). Upper respiratory tract samples, including nasopharyngeal swab (NPS), oropharyngeal swab (OPS), saliva and lower respiratory tract samples such as sputum, are the most widely used specimens for diagnosis of SARS-CoV-2 using RT-qPCR. This study aimed to compare the diagnostic performance of different samples for Coronavirus disease 2019 (COVID-19) detection. It was found that NPS, the reference respiratory specimen for COVID-19 detection, is more sensitive than OPS. However, the application of NPS has many drawbacks, including challenging sampling process and increased risk of transmission to healthcare workers (HCWs). Saliva samples can be collected less invasively and quickly by HCWs with less contact or by own patients, and they can be considered as an alternative to NPS for COVID-19 detection by RT-qPCR. Additionally, sputum, which demonstrates higher viral load can be applied in patients with productive coughs and negative results from NPS. Commonly, after viral RNA purification from patient samples, which is time-consuming and costly, RT-qPCR is performed to diagnose SARS-CoV-2. Herein, different approaches including physical (heat inactivation) and chemical (proteinase K treatment) methods, used in RNA extraction free- direct RT-qPCR, were reviewed. The results of direct RT-qPCR assays were comparable to the results of standard RT-qPCR, while cost and time were saved. However, optimal protocol to decrease cost and processing time, proper transport medium and detection kit should be determined.

## Introduction

**Figure 1:**
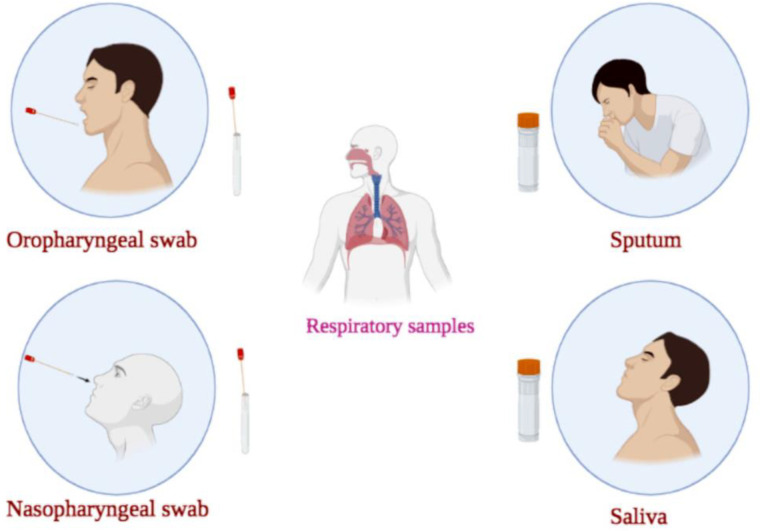
Different respiratory samples for COVID-19 detection. The respiratory tract samples for COVID-19 detection are divided into the upper (nasopharyngeal swab (NPS)/oropharyngeal swab (OPS), NP wash or saliva) and the lower (sputum) parts

Coronavirus disease 2019 (COVID-19) is an acute and high transmissible respiratory disease with severe morbidities and high mortality rates, caused by severe acute respiratory syndrome coronavirus 2 (SARS-CoV-2) (1-3). Real-time quantitative reverse transcriptase-polymerase chain reaction (RT-qPCR) is the most extensively used diagnostic method for detecting SARS-CoV-2 infection, which can be done on different samples (2, 3). The respiratory tract samples for COVID-19 detection are divided into the upper (nasopharyngeal swab (NPS)/oropharyngeal swab (OPS), NP wash or saliva) and the lower (sputum, tracheal aspirate, bronchoscopic brushing or bronchoalveolar lavage fluid (BLF)) parts (Figure 1) (4). Upper respiratory tract sampling is less invasive and decreases the risk of aerosolization and transmission to healthcare workers (HCWs). Therefore, world health organization (WHO) guidelines stated the superiority of upper respiratory tract sampling in ambulant, asymptomatic or mild cases (4). It was found that the sampling from the upper respiratory tract during the first week after illness onset causes a significantly higher viral load. NPS and OPS are the most widely used samples for COVID-19 detection using RT-qPCR. However, the problem of false-negative results in upper respiratory samples taken from asymptomatic individuals or mild cases and the need for repeat sampling and tests still exists (4). Negative results do not rule out COVID-19 infection (2). Although the use of NPS for COVID-19 detection by RT-qPCR is a common method, some studies have applied other samples, including rectal swabs, lower respiratory tract (LRT) and sputum for diagnosis of SARS-CoV-2 (5, 6). Herein, in addition to NPS, OPS, sputum and saliva, mouth rinse/gargle as an alternative sample for SARS-CoV-2 diagnosing using RT-qPCR were discussed. In this study, we aimed to compare the performance of RT-qPCR conducted on different respiratory tract samples.

Quick and early detection of positive cases for COVID-19 infection is the most important aspect of disease control. One approach to achieve control is to train patients to collect their own samples at home and deliver them to medical laboratories for diagnosis. This method causes wider availability with lower costs, prevents close contact in healthcare settings, decreases the risk of exposure to the virus, recognizes asymptomatic but infective carriers and causes focusing of potential medical care on critically ill patients (7, 8). Although, data on the diagnostic performance of self-collected swabs for COVID-19 detection testing are not sufficient, national health service (NHS, United Kingdom) and centers for disease control and prevention (CDC, USA) approved self-collection as an initial diagnostic testing method for diagnosing SARS-CoV-2 (9). Herein, the studies focused on the comparison between self-collected and clinician-collected swabs were also reviewed. 

Usually, three steps before performing quantitative PCR, including 1) purification of total RNA from the sample, 2) elution and possible concentration of the material, and 3) synthesis of complementary DNA (cDNA) from the template RNA are performed (10). Combining RT and PCR using a single reaction kit is common. However, RNA extraction is laborious, expensive, time-consuming and needs manual handling, which may cause experimental errors. Altogether, RNA purification is rate-limiting compared to the downstream RT-qPCR analysis (10, 11). Many studies focused on avoiding RNA extraction in COVID-19 detection (10). In the current study, different methods of RNA extraction free- SARS-CoV-2 RT-qPCR were also reviewed. 

**Nasopharyngeal swab and Oropharyngeal swab**

According to CDC guidelines, NPS and OPS are suitable respiratory specimens for the detection of SARS-CoV-2 RNA (12). NPS and OPS are the most widely used samples for COVID-19 detection using RT-qPCR (13). NPS is collected from the nasopharynx by slightly elevating the tip of the nose, inserting the swab into the nasal cavity and then rotating the swab in the nasopharynx for several seconds. For the collection of oropharyngeal secretions, the tip of the swab must be placed at the posterior oropharyngeal wall, and touching the tongue should be avoided. To absorb respiratory secretion, NPS, as well as OPS, should be left at the appropriate site for a few several seconds. Immediately after sampling, the swabs should be located into transport media, and then the tube caps should be tightened. The swab samples should be kept at 2–8 ºC and immediately submitted to the laboratory (2). It was found that foam swabs have a superior ability to collect the virus compared to polyester swabs and have lower Ct values than polyester swabs. However, due to the high correlation between polyester and foam Ct values, polyester swabs stored in viral transport medium (VTM) or saline can be used as an alternative to foam swabs, especially in times of shortages (14). Nevertheless, NPS as the reference respiratory specimen has some disadvantages such as: requiring close contact between HCWs and patients and also personal protective equipment in sample collection, high risk of virus transmission, not being adequate for serial virus monitoring, being difficult to perform in children, causing discomfort in patients, frequent reflex sneezing or coughing as well as worldwide deficiency of swabs and transport medium (15, 16).

*Comparison between self-collected and clinician-collected swabs*

The studies that compared self-collected and clinician-collected swabs for COVID-19 detection using RT-qPCR were reported and described in Table 1. Some studies reported that the results of RT-qPCR using clinician-collected swabs are comparable to self-collected swabs, especially in cases with higher viral loads (17). Therchilsen *et al.* reported the acceptable agreement and almost equivalent sensitivity (84.2% for self- and 89.5% for HCW-collected samples) between mobile-phone video-instructed self-collected oropharyngeal as well as nasal samples and HCW-collected oropharyngeal samples (9). In another study, the sensitivity and specificity of self-collected midnasal swabs and clinician swabs were reported 80.0% and 97.9%, respectively. Furthermore, false-negative results in patients with low primary viral loads were seen (17). However, Abdollahi *et al.* reported moderate agreement between RT-qPCR results of the NPS and OPS samples obtained by the patients and those collected by the lab technicians (18). Overall, future studies should examine the diagnostic accuracy and cost-effectiveness of self-testing methods in a larger and more heterogeneous cohort of patients (9).

*Comparison between NPS and OPS*

The collection of OPS may be less challenging and needs less training than NPS (12). However, many studies demonstrated that NPS has a significantly higher COVID-19 detection rate, sensitivity, and viral load than OPS (Table 2). In the review article conducted by Mawaddah *et al.*, it was revealed that the viral load and sensitivity of NPS are higher than OPS (4). Many studies recommended the application of NPS for the diagnosis of COVID-19 and monitoring SARS-CoV-2 load (12, 19 and 20). For example, Wang *et al.* demonstrated that 73.1% of NPS positive cases are negative in OPS, indicating that NPS has a higher positive rate than OPS for COVID-19 detection, and OPS may result in a high false-negative rate (13). Another study showed that the COVID-19 detection rate is 10.0% for OPS and 46.7% for NPS, which was significantly more sensitive than OPS (20). Furthermore, Patel *et al*. reported that the absolute sensitivity for NPS and OPS is slightly different when the swabs are collected at ≤7 days after illness onset. However, the sensitivity of NPS and OPS collecting at >7 days after illness onset was 71.4% for OPS and 100.0% for NPS. In addition, the Ct value of NPS was lower than of OPS, indicating more viral load in NPS (12). 

**Saliva samples**

SARS-CoV-2 can be presented in saliva via three potential routes, including upper respiratory tract, blood and infection of the major and minor salivary glands (15). Some studies have suggested the application of saliva samples for COVID-19 detection by RT-qPCR, which compared to swabs, are less invasive, simpler, and can be collected quickly by HCWs with less contact or by own patients or children’s parents. Therefore, the risk of infection can be potentially reduced. In addition, this method is less costly due to the absence of swabs or viral transport media in saliva collection (15). Additionally, saliva can be used as a possible sample for screening and detecting COVID-19 in children (21). It was also found that viral RNA load in saliva samples is stable when stored at room temperature (RT) for 1 day (22) or 2 days (23). Saliva storage at RT did not affect the test sensitivity (24). However, the sensitivity of saliva samples for SARS-CoV-2 detection in literature is variable and further studies in larger cohorts are required. Clear protocols for saliva sample collection may eliminate some of the variations reported in different studies. Furthermore, consistent results of different studies support the clinical application of saliva samples in a healthcare setting (25).

**Mouth rinse/gargle**

The anatomic region in the pharynx gargle is the same as throat swabs. Application of pharynx gargle samples is an accepted method for molecular detection of common respiratory infections. Pharynx gargle samples can be easily self-collected without close contact between patients and HCWs (26). The users are explained to squeeze the contents of the sterile vial containing saline into their open mouth. Next, they are asked to perform a swish/gargle cycle three times and then throw the saline out into a sterile empty container (23). It was reported that detection of viral RNA in saline mouth rinse/gargle is stable after 2 days storage at RT (23).

*Comparison between saliva/gargle and swabs*

Many studies demonstrated that neat undiluted saliva is suitable for self-collection and can be accepted as an alternative to NPS for COVID-19 detection by RT-qPCR in community settings and population-based screening (Table 3). In the study of Babady *et al.*, the overall agreement between saliva samples and OPS was 93%, with a sensitivity of 96.7%. The agreement between saliva and NPS was 97.7%, with a sensitivity of 94.1% (22). Furthermore, Pasomsub *et al.* reported a strong agreement (97.5%) for COVID-19 detection between NPS/throat swab and saliva sample in 200 pairs of the samples (27). 

In the study of Vaz *et al.*, the sensitivity and specificity of saliva were 94.4% and 97.62%, respectively. The overall agreement between the sample pairs of NPS/OPS and saliva was 96.1% (28). Uwamino *et al.* also demonstrated that SARS-CoV-2 detection in supervised collected saliva by patients within 10 days of symptom onset (acute phase) is as accurate as of that using NPSs (24). In the study of Senok *et al.*, the sensitivity and specificity of saliva using NPS as the reference were 73.1% and 97.6%, respectively. The lower sensitivity in this study might be due to performing the study in a screening center, the absence of patients with severe COVID-19 and so, clinical heterogeneity of patients (29). Leung *et al.* demonstrated that the overall rate for SARS‐CoV‐2 detection by RT‐PCR from deep throat saliva (DTS) is comparable to that from NPS samples. However, it is highly required to explain or supervise the procedure of posterior oropharyngeal saliva sampling for patients (30). 

In contrast to many studies that revealed higher sensitivity in NPS than in saliva, some studies demonstrated positive results in saliva not in NPS on the same day, which should be additionally investigated (31). Interestingly, Teo *et al.* demonstrated that saliva is more sensitive than the corresponding NPS. In this study, 62.0%, 44.5%, and 37.7% of saliva, NPS and self-administered nasal (SN) samples were positive. The authors assumed that different test-positive rates in saliva in their study might be the result of using different RT-qPCR kits (32). Reversely, Ku *et al.* demonstrated that self-collected saliva and buccal swabs have a moderate agreement with HCW-collected NPS to detect COVID-19 (33). 

The difference between the results of studies using NPS and saliva may be the cause of viral enrichment in nasal and oropharyngeal secretions, NPS sampling by trained healthcare staff, no evidence for contamination of NPS, higher volume of saliva samples and their pre-processing with dithiothreitol (DTT) before RNA isolation to remove their viscosity (32). As SARS-CoV-2 is likely to be at its highest level in saliva during the first week of infection and NPS continues to be positive even in the convalescent phase, different results may depend on the sampling time (34).

The comparison of the oral rinses and swabs for COVID-19 detection in literature did not lead to a conclusive result (Table 3). For example, Babady *et al.* found that oral rinses are not proper alternative to swab methods. The sensitivity of oral rinse was 63%, and the overall agreement between oral rinse and NP was 85.7% (22). In contrast, in the study of Goldfarb *et al.,* compared to NPS, self-collected saliva and saline mouth rinse/gargle samples, the latter showed the highest user acceptability ratings and diagnostic performance. The sensitivity was 98% for saline mouth rinse/gargle samples and 79% for saliva samples. They attributed the improved recovery of viral RNA in their study to their method (three cycles for mouth rinse followed by gargle) to collect saline mouth rinse/gargle samples (23). In addition, the review article conducted by Mawaddah *et al.* revealed that patient self-collected throat washing has a higher viral load than NPS and OPS and is more sensitive than paired NPS (4).

**Sputum**

A higher level of viral load can be detected in samples from LRT compared to NPS and OPS. Furthermore, positive relation between sputum viral load and severity of COVID-19 as well as the risk of progression was reported (35). However, coughing up is necessary to collect sputum from the lower airways. Therefore, all patients, especially elderly or asymptomatic patients, cannot produce sputum (35). CDC stated that LRT samples, such as sputum, are allowed for patients with a productive cough or under particular situations (*e.g*., invasive mechanical ventilation). Due to the production of aerosols during sputum acquisition, induced sputum was not suggested by the CDC. However, endotracheal sputum aspirates or self-collected sputum (with proper instructions) can be beneficial. Sputum samples can be suitable for patients with a traumatic fracture or anatomic anomaly in the facial/nasal area and patients with productive cough and negative test results using NPS. The presence of mucus in sputum samples is problematic, which can be overcome by CDC guidelines for sputum preparation (36). CDC has suggested pretreating sputum with DTT for COVID-19 detection. In the study of Peng *et al.*, sputum pretreatment using saline, N-acetyl-L-cysteine (NALC), proteinase K (PK), and DTT were compared. A higher COVID-19 detection rate was achieved by pretreatment of sputum samples using PK and DTT compared to their pretreatment with NALC or saline (37). Taken together, PK or DTT pretreated sputum samples can be validated on both manual and automated platforms (36, 37).

*Comparison between sputum and swabs*

The superiority of sputum compared to swabs for COVID-19 detection of SARS-CoV-2 was demonstrated by different studies (Table 4). It was found that the viral load in sputum samples is higher than that in NPS, OPS or throat gargle (4). It was shown that sputum induction might be more suitable than throat swabs for COVID-19 detection in convalescent patients (38). Lin *et al.* evaluated 54 cases of paired specimens of OPS and sputum. They demonstrated that the positive rate of sputum specimens is almost two-fold of throat swabs (39). In another study, He *et al.* compared the COVID-19 detection rate of nasal swabs, throat swabs, feces and sputum. They reported that the highest detection rate and existence time of the viral nucleic acids of COVID-19 is in sputum samples (40). Wu *et al.* also examined the detection of COVID-19 in different samples of 132 patients in Wuhan City. They reported that the positive rate of sputum is higher than NPS (41). 

**Different methods for COVID-19 detection using direct RT-qPCR**

**Figure 2:**
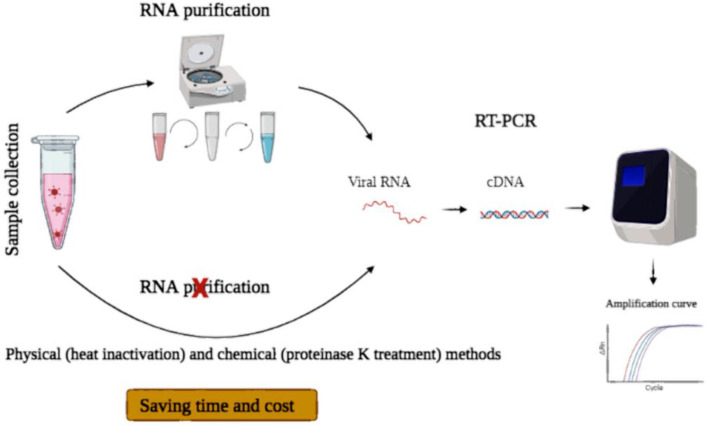
Schematic representation of standard and direct RT-qPCR. In standard RT-qPCR, respiratory samples were collected, and in the case of swabs, they were transferred into a transport medium. Then, viral RNA was extracted, and an RT-qPCR reaction was performed. In direct RT-qPCR, the virus was inactivated by different methods, including heating, proteinase K treatment, pH changing, and then these inactivated samples were used for COVID-19 detection by RT-qPCR. In this process, time and cost were saved

Generally, COVID-19 testing is performed when symptoms are apparent. At that time, viral load is usually high (42). Direct RTqPCR, which eliminates the viral RNA isolation step, is performed faster and simpler than tests requiring an initial RNA extraction step (Figure 2). Therefore, direct RT-qPCR can be used in resource-limited regions with moderate access to RNA purification kit. This approach will cause widespread testing in these regions while saving time and cost (11). However, due to the presence of inhibitors of RT-qPCR in biological samples and RNA loss caused by heating and/or RNases, the use of pre-treated samples directly in RT-qPCR is challenging (43). Therefore, many studies focused on developing different direct RT-qPCR methods to obtain equivalent results to the established method that involves RNA extraction (Table 5).

Many kit-free protocols consider physical (heat inactivation) or chemical (PK treatment) methods. Heat inactivation of SARS-CoV-2 can be performed at 60 ºC for 32.5 min, at 80 ºC for 3.7 min, and at 100 ºC for 0.5 min. Application of internal control for real-time RT-qPCR reaction confirmed that these temperatures, which are used during amplification by real-time RT-qPCR, do not affect the quality of genetic materials of the samples. PK prevents contamination from nucleic acid preparations by protein digestion and inactivating nucleases that can degrade DNA or RNA during purification (44). In the study of Alcoba-Florez *et al.*, three different heating treatments (70 ºC incubation for 10 min), including direct NPS viral transmission medium (VTM) heating before the RT-qPCR, in a formamide-EDTA (FAE) buffer and in an RNAsnap^TM^ buffer were evaluated. Direct heating without additives led to the best results and was highly consistent with the COVID-19 detection by a standard method in almost half of the time (45). In the study of Hasan *et al.*, the sensitivity, specificity and accuracy of the optimized RNA extraction-free protocol, which included 4- fold specimen dilution, incubation of specimens at 65 ºC for 10 min followed by application of TaqPath™ 1-Step RT-qPCR master mix were 95%, 99% and 98.5%, respectively. They attributed equivalent results from the direct and standard RT-qPCR to low heat strategy, proper dilution of inhibitory substances and higher sensitivity of TaqPath™ 1-Step RT-qPCR Master Mix (43). Smyrlaki *et al.* performed RT-qPCR directly on heat-inactivated samples and sample lysates. In this study, after placing the clinical samples in a transport medium, viral particles were inactivated by heating or direct lysis in detergent/chaotropic reagents. Then, the inactivated samples were applied in downstream RT-qPCR diagnostic reactions. Inactivation at 95 ºC for 5 min demonstrated improved (reduced) Ct value of heat-inactivated direct RT-PCR (hid-RT-PCR) compared to that at 65 ºC for 30 min. Due to the cleavage of RNA into shorter fragments by heat inactivation, it is very important to consider primer and probe in hid-RT-PCR. It was found that the primer-probe set with the shortest amplicon is the best in hid-RT-PCR.

Furthermore, because of possible inhibition from the sample, it is necessary to optimize the amount of sample input in hid-RT-PCR. It was demonstrated that an input of 1–4 μL sample in a 20 μL RT-qPCR reaction is optimal. A strong correlation between Ct values of extracted and heat-inactivated samples was observed. However, higher Ct values for hid-RT-PCR on frozen samples compared to fresh RNA eluates of the same samples were reported (10). In the study of Bruce *et al.*, the best sensitivity for COVID-19 detection using direct RT-qPCR was obtained when 3 μL of swab diluent was applied. In this study, 92% of positive NP samples examined by traditional RT-qPCR were positive by direct RT-qPCR approach without RNA extraction step. Only samples with very low levels of viral RNA were missed. The sensitivity of 95% was achieved when samples with a clinical Ct at or below 32 were examined by direct RT-qPCR approach. Therefore, the sensitivity of direct RT-qPCR method would be sufficient to detect the patients most possible to be infectious (11). Beltr´an-Pavez *et al.* also demonstrated that the sensitivity over 90% is achieved when direct RT-qPCR without prior RNA extraction is applied for samples with Ct values lower than 30; while, sensitivity is decreased to 19-40%, when Ct values of the samples are higher than 30 (42). Kriegova *et al. *developed DIOS-RT-qPCR assay, in which RNA purification step was excluded, heat inactivation of SARS-CoV-2 (75 ºC for 10 min) was applied, and speed was increased through application of fast enzymes that have a high tolerance to inhibitors. In this assay, enzymes that endured a large volume of the swab (14 μL of swab diluent) were used. In DIOS-RT-qPCR, using large volumes of the swab made it possible to obtain comparable sensitivity to methods based on RNA isolation. However, in other direct RT-qPCR protocols, in which usually 3-5 μL of samples were applied, moderate sensitivity in samples with low viral loads was achieved (46). 

In addition, heat inactivation (95 ºC for 30 min) of saliva samples, adding TBE buffer and Tween 20, followed by RT-qPCR, was introduced as the safest and most streamlined protocol by the University of Illinois. It was found that heating at 95 °C for 30 min unlike heating at ~60 ºC for 30 min, which was applied by many protocols, can inactivate the inhibitory component of RT-qPCR presents in saliva. Adding Tween-20 may help open the viral capsid and liberation of RNA to supply enough template for RT-qPCR detection. The primary examination of clinical samples using unoptimized protocol demonstrated the sensitivity and specificity of 88.9% and 98.9%, respectively (34).

Chomczynski *et al.* developed a method, named alkaline-glycol processing (AG processing), in which a biological sample of COVID-19 patients, such as saliva or a swab-collected suspension, was incubated in an alkaline-glycol solution (pH 12.2 to 12.8) at RT for 5 to 30 min and then evaluated for the presence of a viral RNA by direct RT-qPCR. It was found that concentrated polyglycols in alkaline aqueous solution lyses viruses and decrease the effect of inhibitors. The LOD was 300 viral copies per mL of initial saliva specimen (47). In another direct RT-qPCR test for detecting SARS-CoV-2 RNA, named DIRECT-PCR, saliva was treated with DTT and inhibitor-resistant enzymes. As few as six RNA copies per reaction of N gene can be detected from respiratory samples such as sputum and nasal exudate in less than an hour by this one-step assay, in which viral lysis, reverse transcription, amplification, and detection are performed in a single-tube homogeneous reaction (48).

In another approach, NPSs were treated with PK (3 μg/μL, 56 ºC for 10 min) and thermal shock (98 ºC for 5 min followed by 4 ºC for 2 min). The concordance between the samples extracted using an RNA extraction commercial kit and the established in-house method was 100%. There was no significant difference between the RNA extraction method using a commercial kit and in-house PK, followed by thermal shock (44). In the study of Michel *et al.*, dry swabs were resuspended in normal saline, treated with PK, centrifuged and incubated in a dry thermal block at 56 ºC for 3 min and then at 95 ºC for 3 min to inactivate the proteinase. The developed method, named COVID-quick-DET, demonstrated a sensitivity of 94.6% compared to RNA extraction kit-based methods. COVID-quick-DET provided RNA for quantitative PCR analysis in appx. 90 min *vs.* 280 min kit-based per 100 samples and its detection limit was Ct value around 31–33 (49).

Overall, it is required to optimize direct RT-qPCR protocol in order to reduce cost and processing time. Therefore, examining adequate samples in developing countries will be possible using the optimized RT-qPCR protocol (44). Furthermore, the consequence of an extraction-free method strongly relates to proper transport medium and detection kit. As the efficiency of the PCR may be reduced by extraction-free methods, it is very important to select a proper sensitive RT-qPCR kit (50).

**Table 1:** Comparison between self-collected swabs and clinical-collected swabs for diagnosing SARS-CoV-2 using RT-qPCR

**Country**	**No. of samples**	**Type of samples**	**Sensitivity**	**Specificity**	**Overall agreement**	**Main Findings**	**Ref.**
Washington	185	Unsupervised home self-collected midnasal swab	80.0%	97.9%	Self- *vs*. clinician-collected swabs: Cohen kappa: 0.81	Substantial agreement between technician-collected and patient-collected swabs	(17)
		Clinician-collected NPS					
Denmark	109	Self-collected OP swabs	84.2%	-	Self- *vs*. clinician-collected swabs: Cohens kappa: 0.82 (*p *< 0.001)	Acceptable agreement and almost equivalent sensitivity in technician-collected and patient-collected swabs	(9)
		Clinician-collected OPS	89.5%				
Tehran, Iran	50	Patient-collected NPS/OPS			Self- vs. clinician-collected swabs: 76% Cohen kappa value: 0.49 (*P *= 0.001)	Lab technician-collected NP or OPS cannot be replaced by patient-collected ones	(18)
		Technician-collected NPS/OPS					

NPS: nasopharyngeal swabs; OPS: oropharyngeal swabs; RT-qPCR: quantitative reverse transcription polymerase chain reaction;

SARS-CoV-2: severe acute respiratory syndrome coronavirus 2.

**Table 2:** Comparison between OPS and NPS for diagnosing SARS-CoV-2 using RT-qPCR

**Country**	**Type of samples**	**No. of samples**		**Detection rate**	**Sensitivity**	**Ct value**	**Other data**		**Main Findings**	**Ref.**
China	NPS and OPS	353		NPS: 19%	-	-	Correlation between NPS and OPS: Kappa = 0.308		Higher positive rate of NPS than OPS for COVID-19 detection OPS may result in high false negative rate Poor Correlation between NPS and OPS	(13)
				OPS: 7.6%						
				Combined: 21.5%						
China	NPS and OPS	120		NPS: 46.7%	NPS: 98.3%	NPS: 37.8	Median duration of COVID-19 detection	Maximum duration of COVID-19 detection	Significant higher sensitivity of NPS than OPS	(20)
							NPS: 25days	NPS: 41 days		
				OPS: 10.0%	OPS: 21.1%	OPS: 39.4	OPS: 20.5 days	OPS: 39 days		
Georgia, USA	NPS and OPS	146	Collected ≤7 days after illnessonset	NPS: 15.1%	NPS: 88%	NPS (N1): 24.3 (N2): 25.0	-		The sensitivity of NPS and OPS collecting at >7 days after illness onset was more different than that collected at ≤7 days after illness onset 95.2% concordant results between NPS and OPS collected ≤7 days after illness onset	(12)
						OPS (N1): 29.9 (N2): 31.4				
				OPS: 14.4%	OPS: 84%					
			Collected >7 days after illnessonset	NPS: 23.7%	NPS: 100%	-				
				OPS: 17.0%	OPS: 71.4%					

NPS: nasopharyngeal swabs; OPS: oropharyngeal swabs; RT-qPCR: quantitative reverse transcription polymerase chain reaction; SARS-CoV-2: severe acute respiratory syndrome coronavirus 2.

**Table 3:** Comparison between saliva/ gargle and swabs for COVID-19 detection using RT-qPCR

**Type of samples**	**Country**	**No. of Samples**	**Sensitivity**	**Specificity**	**Overall agreement**	**NPV**	**PPV**	**Ct values**	**Main Findings**	**Ref.**
NPS and saliva	Japan	196	-	-	NPS and saliva: 96.4% Kappa coefficient: 0.883				The same accuracy of saliva and NPS collected in an acute phase for detection of SARS-CoV-2 using RT-qPCR Saliva sample storage at room temperature did not affect the test results	(24)
NPS/OPS,saliva and OR	USA	570	Saliva compared with NPSs: 94.1%	Saliva compared with NPSs: 98.6%	NPS and saliva: 97.7% Kappa coefficient: 0.93	-	-	Mean Ct (N2) in NPS and OPS: 22.6 and 28.6	Saliva is an acceptable alternative to NPSs for SARS-CoV-2 RNA detection by RT-qPCR No changes in viral loads of saliva over 24 hours storage at both RT and 4 ˚C	(22)
			Saliva compared with OPSs: 96.67%	Saliva compared with OPSs: 91.43%	OPS and saliva: 93% Kappa coefficient: 0.84			Mean Ct (N2) in saliva: 27.9		
			OR compared with NPS: 63.6	ORcompared with NPS: 96.9%	ORand NPS: 85.7% Kappa coefficient: 0.65			Mean Ct (N2) in OR: 29.9		
NPS and saliva	United Arab Emirates	401	Saliva compared to NPS: 73.1 %	Saliva compared to NPS: 97.6%	NPS and saliva: 96.0% Kappa coefficient: 0.68	98.1%	67.9%		Good diagnostic accuracy and feasibility of utilization of specimen without transport media for Saliva	(29)
NPS/OPS and saliva	Brazil	155	Saliva compared to NPS/OPS: 94.4%	Saliva compared to NPS/OPS: 97.62%	NPS/OPS and saliva: 96.1%	95.35%	97.1%		High overall agreement between NPS/OPS and saliva	(28)
NPS/OPS and saliva	Thailand	150	Compared to NPS/throat swabs: 84.2%	Compared to NPS/throat swabs: 98.9%	NPS/OPS and saliva: 97.5% Kappa coefficient: 0.851	98.4%	88.9%	Median Ct (ORF1ab and N) in salvia: 32.7 and 31.8	High sensitivity and comparable performance of saliva to NPS/throat swab Strong agreement between NPS/throat swab and saliva sample	(27)
								Median Ct (ORF1ab and N) in NPS/throat swabs: 32 and 30.5		
NPS/OPS and saliva	Singapore	42	-	-	NPS and saliva: 69.0%	52.4%	95.2%	Ct in saliva: 25.77 ± 5.60	Moderate agreement between self-collected saliva/buccal swabs and HCW-collected NPS	(33)
								Ct in NPS: 22.95 ± 6.03		
NPS, saliva and gargle	Canada	50	Saline mouth rinse/gargle: 98%	-	-				Demonstrating higher combined user acceptability ratings and analytical performance for saline mouth rinse/gargle samples than saliva and HCW-collected NPS	(23)
			Saliva sample: 79%							
NPS and DTS	China	95	-	-	NPS and DTS: 78.9% Kappa coefficient: 0.58				Equivalent overall performance of DTS to that of the NPS	(30)

DTS: deep throat saliva; HCW: healthcare workers; NPS: nasopharyngeal swabs; NPV: negative predictive value; OPS: oropharyngeal swabs; OR: Oral rinse; PPV: positive predictive value; RT: room temperature; RT-qPCR: quantitative reverse transcription polymerase chain reaction; SARS-CoV-2: severe acute respiratory syndrome coronavirus 2.

**Table 4:** Comparison between sputum and swabs for COVID-19 detection by RT-qPCR

**Country**	**No. of Samples**	**Type of samples**	**Detection rate**	**Prolonged day**	**Main Findings**	**Ref.**
China	54	Throat swabs	44.2%	-	Significant higherCOVID-19 detection rates from sputum specimens than those from throat swabs	(39)
		Sputum	76.9%	-		
China	20	Sputum	95% (19/20)	42.8 ± 4.2	More prolonged survival period of SARS-CoV-2in sputum specimens from COVID-19 patients	(40)
		Throat swabs	**-**	32.0		
		Nasal swabs	**-**	24.0		
China	132	Sputum	48.68% (148/304)	**-**	Higher detection rate in sputum than NPS	(41)
		NPS	38.13% (180/472)	**-**		

COVID-19: Coronavirus disease 2019; NPS: nasopharyngeal swabs; RT-qPCR: quantitative reverse transcription polymerase chain reaction; SARS-CoV-2: severe acute respiratory syndrome coronavirus 2.

**Table 5:** Different direct RT-qPCR methods for diagnosing SARS-CoV-2 RNA

**Sample type**	**No. of sample**	**Inactivation method**	**Sensitivity**	**Specificity**	**Overall agreement**	**Detection limit**	**Detection time**	**Ref.**
NPS	597	Adding Lysis Buffer** + **Heating at 65 ˚C for 30 min	96.0%	99.8%	98.8%	ORF1: 0.009 TCID_50_/mL E: 0.003 TCID_50_/mL	**-**	(10)
NPS	Pooled patient sample	Heating at 70 °C for 5 min	95% on samples with a clinical Ct at or below 32	**-**	**-**	1.7×10^4^copies RNA/mL	-	(11)
Saliva	-	Buffer Dilution+ heating at 95 °C for 30 min+ Tween-20	88.9%	98.9%	-	1,000 copies of SARS-CoV-2 virus per mL of saliva spiked with γ-irradiated SARS-CoV-2	-	(34)
NPS	80	Heating at 95 ºC for 5 min	Over 90%	-	95%	-	Saving time: 2 h compare to standard RT-qPCR	(42)
Nasopharyngeal specimens positive for SARS-CoV-2 and other coronaviruses collected in universal viral transport (UVT) medium	132	4- fold specimen dilution+ incubation at 65 ˚C	95%	99%	98.5%	6,600 copies per mL of a viral suspension spiked with synthetic SARS-CoV-2 RNA	-	(43)
Nasal and pharyngeal swab	78	PK (3 μg/μL, 56 ˚C for 10 min) and thermal shock (98 ˚C for 5 min followed by 4 ˚C for 2 min)	-	-	100%	-	Saving time: 30 min compare to standard RT-qPCR	(44)
NPS	90	Incubation at 70 ˚C + additives in formamide-EDTA (FAE) buffer and RNAsnap^TM^ buffer	87.8%	100%	99.9%	-	Saving time: 1-1.2 h compare to standard RT-qPCR	(45)
NPS nasalSwab	-	Heating at 75 ˚C for 10 min	-	-	98%	550 virus copies/mL of swab	Less than one hour	(46)
Saliva	-	Incubation in an alkaline-glycol solution samples (pH 12.2 to 12.8)	32-foldincrease in detectionsensitivity by AG processing	-	-	300 copies per mL of saliva	-	(47)
Sputum and nasal exudate	-	Dithiothreitol (Sputasol) + ribonuclease inhibitor	-	-	-	12 copies per PCR reaction volume	36 min	(48)
Throat swabsand other materials from the respiratory tract	-	PK treatment + repetitive heating steps (56 ˚C for 3 min and then at 95 ˚C for 3 min)	94.6%	-	-	5–10genome per mL	90 min	(49)

Ct: cycle threshold; NPS: nasopharyngeal swabs; PK: Proteinase K; RT: room temperature; RT-qPCR: quantitative reverse transcription polymerase chain reaction; UVT: universal viral transport.

## Conclusion

Earlier sampling after symptom onset would enhance the COVID-19 detection rate. However, COVID-19 detection by different sampling methods varied irrespective of the duration of symptoms (16). A systematic review and meta-analysis studied the positive COVID-19 detection rate in various clinical specimens using RT-qPCR. The most positive detection rate was shown in the BLF sample (91.8%), while no virus was found in urine samples. The order of positive detection rate in other samples was as follows: rectal swabs (87.8%), LRT (71.3%), sputum (68.1%), NPS (45.5%), feces (32.8%), OPS (7.6%) and blood samples (1.0%) (5). In the study of Wu *et al.*, some of the negative results related to the respiratory tract (sputum and NPS) was positive in the digestive tract (fecal and anal swab), demonstrating that the clearance time of COVID-19 in the digestive tract is later than that in the respiratory tract and the virus can be transmitted by fecal route (41). Furthermore, prolonged SARS‐CoV‐2 positivity in anal/rectal swabs and stool samples were reported in COVID‐19 patients after negative conversion in nasopharyngeal RT‐PCR test (6). Totally, rectal swab is suggested for COVID‐19 detection. However, due to high viral load in upper respiratory tract during the first week after illness onset, many studies focused on upper respiratory tract samples. In a systematic review and meta-analysis conducted on 3442 respiratory tract specimens, it was revealed that significantly higher rate of SARS-CoV-2 RNA detection is achieved using sputum sample compared to NPS, and OPS demonstrated the lowest positive rate (16). However, access to sputum in COVID‐19 patients due to dry cough as usual clinical presentation of COVID‐19 is limited. Overall, to date the literature demonstrated that NPS remains the gold standard in many parts of the world in comparison to other alternative samples (16, 51). A meta-analysis by Czumbel *et al.* showed that the sensitivity of NPS (98%) is higher than that of saliva (91%). However, due to lack of significant difference, saliva sample can be used in RT-qPCR test as promising alternative to NPS for COVID-19 detection (52). Another systematic review and meta-analysis also confirmed that the diagnostic performance of saliva is a little lower than NPS. The positive detection rate for saliva samples was increased by self-collection, coughing or deep throat saliva, and avoiding food, drink, or toothbrushing and was decreased by saliva collection after 7 days post symptom onset. However, in both cases the difference was not significant (51). Saliva samples should serially be collected early morning before brushing and eating for quality assurance (31). Furthermore, considering the promising results of sample combination, simultaneous application of NPS and throat wash or saliva may help to resolve the problem of false negative results and reduce the spread of this pandemic. More studies considering different sample combinations, precise details of collection method including the shape and material of swabs, sample processing methods (dilution, extraction, storage, transport) would be helpful for developing and scale up of SARS-CoV-2 testing (51).

Usually, viral RNA is purified from a patient sample, and then RT-qPCR is performed to diagnose SARS-CoV-2 RNA. Direct RT-qPCR assays, in which initial RNA purification steps are eliminated, are faster and simpler than standard RT-qPCR tests. Several direct RT-qPCR diagnostic tests using heat inactivation, pH change or proteinase K treatment have been developed (44, 47). Overall, time and cost were significantly saved through RNA-extraction-free protocols, which were comparable with established PCR-based testing pipelines (10). Additionally, working with inactivated SARS-CoV-2 samples in direct-RT-qPCR would permit to apply patient samples in a biosafety level (BSL)-2 environment instead of a BSL-3 (46). However, the optimal protocol to decrease cost and processing time should be determined. Additionally, a proper transport medium and detection kit should be selected in the direct RT-qPCR method to achieve comparable results to standard RT-qPCR (44).
